# Dietary fat intake and quality of life: the SUN project

**DOI:** 10.1186/1475-2891-10-121

**Published:** 2011-11-02

**Authors:** Cristina Ruano, Patricia Henriquez, Maira Bes-Rastrollo, Miguel Ruiz-Canela, Cristina López del Burgo, Almudena Sánchez-Villegas

**Affiliations:** 1 Department of Clinical Sciences. University of Las Palmas de Gran Canaria, Spain; 2 Department of Preventive Medicine and Public Health, University of Navarra, Spain

**Keywords:** Dynamic cohort, Fatty acids intake, SF-36 Health Survey, Mental quality of life, Physical quality of life

## Abstract

**Background:**

Few studies have related nutritional factors with quality of life in healthy populations. The purpose of the study was to assess whether dietary fat intake is associated to mental and physical quality of life.

**Methods:**

This analysis included 8,430 participants from the SUN (Seguimiento Universidad de Navarra) Project. The intake of saturated fatty acids (SFA), polyunsaturated fatty acids (PUFA), trans unsaturated fatty acids (TFA), and monounsaturated fatty acids (MUFA) was assessed through a 136-item food frequency questionnaire at baseline. Quality of life was measured with the SF-36 Health Survey after 4 years of follow-up. Generalized Linear Models were fitted to assess the regression coefficients (b) and their 95% confidence intervals (95% CI) for the 8 domains of the SF-36 according to successive quintiles of each kind of fatty acids intake.

**Results:**

The multivariate-adjusted models revealed a significant inverse association for SFA intake (in quintiles) and two of the physical domains (physical functioning and general health). E.g. for general health domain: (highest quintile of intake (Q5) vs. lowest quintile (Q1), b = -1.6; 95% CI = -3.1, -0.1. General health also showed a dose-response relationship (p for trend < 0.05). For TFA intake (in quintiles), a significant inverse association was found for most of the mental domains (vitality, social functioning and role emotional). E.g. for vitality domain (Q5) vs. (Q1), b = -2.0, 95% CI = -3.4 to -0.6. We also found an inverse association between TFA intake and the bodily pain domain: (Q5 vs. Q1), b = -2.6; 95% CI = -4.4 to -0.8, with a statistically significant dose-response relationship (p for trend < 0.05). Except for TFA intake and the mental domains, the rest of the associations were attenuated when we repeated the analysis adjusting for adherence to the Mediterranean diet.

**Conclusions:**

A detrimental relationship between TFA intake at baseline and most of the SF-36 mental domains measured 4 years later were found, whereas weak inverse associations were found for SFA intake and some physical domains.

## Background

Quality of life is a broad concept that relates to all aspects of human life. Quality of life questionnaires have become an efficient way of gathering data about people functioning and well being. Also health status measures have been shown to be a powerful predictor for chronic diseases and mortality over the long term in clinical practice [1,2]. Moreover, population ageing has fostered the general concern for obtaining a better health-related quality of life [3].

Epidemiological studies have shown the influence of different factors such as smoking, obesity, or crash injuries on quality of life [4-7]. However, few longitudinal studies have analyzed the influence of diet on the quality of life of healthy populations [8-10]. Several studies have found that the adherence to a Mediterranean dietary pattern rich in fruits and vegetables, legumes, fish and olive oil, was associated with higher scoring for self-perceived health [11,12]. On the other hand, some studies have shown the detrimental effects on health of a "Western-type" dietary pattern rich in processed and red meats, refined grains and commercial baked goods [13-16]. The Western dietary pattern is the common diet in Northern Europe and the USA. In recent years, however, southern European countries, which used to eat a traditional Mediterranean diet, have also been adopting a more Western-style diet. Beyond the health effects of an overall Western dietary pattern, little is known about the health effects of the diet's specific elements, such as major fat sources, on quality of life.

There has been a dramatic change in the sources of fat intake in the general population. This change mainly consists in replacing polyunsaturated (PUFA) or monounsaturated fatty acids (MUFA) with saturated fats (SFA) and trans unsaturated fats (TFA). Usually, PUFA [17] and olive oil (OO) [18-20] have been considered as healthy lipids because they reduce the incidence of cardiovascular disease (CVD). In contrast, SFA and especially TFA are known risk factors for CVD [21]. There are some reasons to expect that CVD share some common determinants, [22] endothelial dysfunction and high levels of pro-inflammatory cytokines, with other pathologies like obesity, insulin resistance and neuropsychological disorders. All of these physiological processes may have also an influence on physical and mental quality of life in healthy population.

From a public health perspective, it is necessary to identify the nutritional factors that could increase or decrease the quality of life and health status of the population. The aim of this study was to assess the association between dietary fat intake and self-perceived health-related mental and physical quality of life in a Mediterranean cohort, the SUN Project.

## Methods

### Study population

The "Seguimiento Universidad de Navarra'' (SUN) Project is an ongoing, multipurpose, dynamic cohort of university graduates conducted in Spain and started in December 1999. As a dynamic cohort, the recruitment of the participants is permanently open. The study methods and the cohort profile have been published in detail elsewhere [23,24].

Information on exposures and outcomes is gathered by postal mail or web-based questionnaires collected biannually. Participants answer a baseline questionnaire assessing multiple exposures such as nutritional habits, physical exercise, medical conditions and other risk factors. Every two years they answer the follow-up questionnaires assessing changes in exposures and new events of interest.

Up to November 2010, 15,089 participants had responded to the baseline and to the 4-year follow-up questionnaire. As the recruitment is permanently open, with approximately 2,000 new participants each year, only those entering the cohort before 2005 could be followed-up for 4 years. The overall retention rate is 92%. From those participants who were assessed after 4-year (15,089), we excluded those who reported extremely low or high values for total energy intake (less than 800 Kcal/day or more than 4,000 Kcal/day in men and less than 500 Kcal or more than 3,500 Kcal/day in women (n = 1,489), those who reported dyslipidemia and cardiovascular disease at baseline (n = 2,924), and those without or with incorrect data regarding quality of life (n = 2,246). Finally, 8,430 participants were included in this analysis.

The study was approved by the Human Research Ethical Committee at the University of Navarra. Voluntary completion of the first questionnaire was considered to imply informed consent. Our Institutional Review Board specifically approved this consent process.

### Exposure assessment

Dietary intake was assessed using a semi-quantitative food frequency questionnaire (136 food items) included at baseline [25]. Validity and reproducibility of this questionnaire has recently been re-evaluated [26]. It showed reasonably good validity for assessing fat intake (energy-adjusted intraclass correlation coefficients for different types of fats versus four 3-day food records ranged from 0.49 to 0.75) [27]. Nutrient intakes of 136 food items were calculated as frequency multiplied by nutrient composition of specified portion size for each food item, using an ad hoc computer program developed for this purpose. A trained dietician updated the nutrient data bank using the latest available information from the food composition table for Spain [28,29].

Baseline intake of every dietary fat: MUFA, n-3 PUFAs, n-6 PUFAs, SFA and TFA were analyzed as quantitative variables (grams per day) and categorized into quintiles. We also analyzed as quantitative variables several culinary fats like seed oil, butter, margarine, and olive oil and we categorized them into quintiles. Adjustments were made for total energy intake by using the residuals method proposed by Willet [30]. The ratio n-3/n-6 was also computed [31].

### Outcome assessment

Quality of life was assessed after a 4-year follow-up with the validated Spanish version of the SF-36 Health Survey. The SF-36 is a general health scale widely used and thoroughly validated [32]. The questionnaire contains 36 items that measure eight multi-item parameters of health status: physical functioning, role limitations due to physical health problems (role-physical), bodily pain, general health perceptions, vitality, social functioning, role limitations due to emotional problems (role emotional) and mental health. Domains 1 to 4 of the questionnaire deal with physical aspects, while domains 5 to 8 measure psychological features. For each parameter, scores were coded, summed and transformed to a scale from 0 (the worst possible condition) to 100 (the best possible condition). For example, for bodily pain a score of 100 means a complete tolerance to pain.

### Covariate assessment

The baseline assessment also gathered information on socio-demographic variables (e.g. sex, age, marital status and employment status), anthropometric variables (*e.g*., weight and height), lifestyle and health-related habits (*e.g*., smoking status), and medical history (*e.g*., chronic diseases) [24]. Self-reported body mass index (BMI) was calculated as weight (in kilograms) divided by the square of height (in meters). Self-reported anthropometrics were previously been validated in a subsample of the cohort [33].

At baseline, participants also completed a validated physical activity questionnaire that collects information about 17 activities [34]. Leisure-time activities were computed by assigning an activity metabolic equivalent (MET) score to each activity, multiplied by the time spent in each activity and summing up all activities [35].

Information about alcohol intake was obtained through the semi-quantitative food frequency questionnaire included in the baseline questionnaire.

Adherence to the Mediterranean diet was assessed combining 8 items (fruits and nuts, vegetables, fish, legumes, cereals, meat and meat products, dairy and alcohol intake) [36]. To avoid overlapping with our main exposure, we excluded the ratio MUFA/SFA item from this score (the actual range in our study was from 0 to 8).

### Statistics

Generalized Linear Models were used to assess the relationship between quintiles of total fat and specific dietary fatty acids intake and each domain of the SF- 36 Health Survey, using always the lowest quintile as the reference. Regression coefficients (b) and their 95% confidence intervals (95% CI) were calculated. Tests of linear trend across successive quintiles were conducted by assigning the medians to each quintile and treating this consumption as a continuous variable.

Age (years, continuous), sex, BMI at baseline (Kg/m^2^), total energy intake (Kcal/day, continuous), physical activity during leisure time (METS- h/week, continuous) and smoking (never, ex smokers and current smokers) were considered as potential confounders in all the models. To further reduce potential confounding, all others fatty acids not considered as exposure variable were also include as covariates in the models. Finally, additional adjustments were made to take into account Mediterranean diet-pattern adherence.

The SPSS software package for Windows version 18.0 (SPSS Inc., Chicago, IL) was used for statistical analyses.

## Results

The main characteristics of the study population according to extreme quintiles of SFA, TFA, MUFA and PUFA intake are shown in Table 1. Younger participants, men and current smokers were more likely to belong to the highest level of SFA and TFA intake (Q5). Participants in Q5 were also less active compared to those in the lowest quintile (Q1). The adherence to the Mediterranean diet score decreased across quintiles of SFA, TFA, MUFA and PUFA intake. This decrease in the score is lower for quintiles of MUFA and PUFA intake.

**Table 1:** Baseline characteristics* according to extreme quintiles of specific types of fat intake in the SUN project.

Characteristic	Saturated fat		*Trans *unsaturated fat		MUFA fat		PUFA fat	
	Q1	Q5	Q1	Q5	Q1	Q5	Q1	Q5
Sex (% men)	37.7	41.1	34.5	43.8	42.9	28.8	36.8	38.8
Age at baseline (y)	39.4 (11.9)	34.6 (9.9)	38.9 (11.6)	35.1 (10.2)	38.4 (12.0)	36.2 (10.6)	37.8 (11.2)	35.3 (10.9)
BMI (kg/m^2^)	23.4 (3.3)	23.0 (3.3)	23.2 (3.3)	23.0 (3.3)	23.3 (3.2)	23.0 (3.3)	23.1 (3.3)	22.9 (3.3)
Smoking:								
Ex smoker (%)	30.4	22.7	30.6	22.0	26.9	29.2	29.1	26.0
Current (%)	19.9	25.3	20.3	23.5	19.7	26.0	21.6	23.2
Leisure time physical activity (Mets-h/wk)	23.6 (25.9)	19.3 (24.1)	23.6 (25.8)	19.1 (21.8)	23.4 (26.4)	19.0 (21.7)	23.2 (27.1)	18.8 (21.3)
Total energy intake (Kcal/d)	2537 (581)	2495 (616)	2616 (551)	2508 (594)	2534 (577)	2503 (610)	2617 (517)	2519 (616)
Mediterranean Dietary Score (0-8)	4.9 (1.4)	2.8 (1.4)	4.8 (1.4)	2.9 (1.5)	4.5 (1.5)	3.4 (1.6)	4.3 (1.6)	3.8 (1.6)
Total fat intake (g/d)*	80.4 (15.9)	113.7 (13.7)	87.8 (19.3)	107.4 (15.1)	77.0 (12.6)	118.5 (12.9)	84.1 (17.4)	108.7 (16.1)
Saturated fat intake (g/d)*	22.8 (4.2)	45.5 (6.4)	25.8 (6.8)	42.0 (8.4)	26.4 (6.3)	39.2 (9.3)	31.0 (9.8)	34.8 (8.4)
Monounsaturated fat intake (g/d)*	34.5 (9.4)	48.4 (8.5)	38.5 (11.6)	45.0 (8.6)	29.6 (4.6)	56.0 (7.3)	35.2 (8.7)	46.0 (10.2)
Polyunsaturated fat intake (g/d)*	12.9 (5.1)	14.6 (4.3)	13.5 (5.3)	14.3 (4.1)	11.6 (4.2)	16.2 (4.9)	9.0 (1.7)	20.5 (4.0)
*Trans *unsaturated fat intake (g/d)*	0.6 (0.3)	1.5 (0.6)	0.4 (0.2)	1.7 (0.4)	0.8 (0.4)	1.2 (0.6)	0.9 (0.6)	1.0 (0.5)

*Continuous variables are expressed as the mean and (standard deviation). Categorical variables are expressed as percentages.

MUFA: Monounsaturated fatty acid. PUFA: Polyunsaturated fatty acid. Q1: First quintile (lowest intake). Q5: Fifth quintile (highest intake)

Table 2 shows the multivariate regression coefficients (b) and their 95% CI for the mental domains of the SF-36 according to baseline SFA and TFA intake (in quintiles). The multivariate-adjusted model revealed a significant inverse association between TFA intake and most of the mental domains (vitality, social functioning and role emotional). For example: b = -2.0, 95% CI = -3.4 to -0.6 (Q5 vs. Q1), for the vitality domain. Moreover a statistically significant dose-response relationship (p for trend < 0.05) was found for each domain except for mental health. No association was found for SFA intake and the mental domains.

**Table 2:** Regression coefficients (b) and 95% Confidence Intervals (CI) for the association between baseline saturated (SFA) and trans unsaturated (TFA) fatty acid intake and the SF-36 mental domains after 4-y follow-up.

	Baseline saturated fatty acid intake					
SF-36 mental scores after 4 years of follow-up	Q1	Q2	Q3	Q4	Q5	p linear trend
Energy-adjusted SFA intake (g/day) (median)	22.8	29.7	33.5	37.3	45.5	
**Vitality**						
Multivariate adjusted model (1)	0 (ref.)	-0.7 (-1.8, 0.5)	-0.2 (-1.4, 1.0)	-0.2 (-1.5, 1.1)	-1.4 (-2.9, 0.1)	0.166
Additionally adjusted for MD (2)	0 (ref.)	-0.4 (-1.6, 0.7)	0.2 (-1.0, 1.4)	0.2 (-0.9, 1.7)	-0.7 (-2.2, 0.9)	0.682
**Social functioning**						
Multivariate adjusted model (1)	0 (ref.)	0.3 (-0.7, 1.3)	1.0 (-0.02, 2.1)	0.6 (-0.5, 1.6)	-0.3 (-1.7, 1.0)	0.779
Additionally adjusted for MD (2)	0 (ref.)	0.3 (-0.7, 1.3)	1.1 (-0.09, 2.2)	0.7 (-0.5, 1.9)	-0.6 (-1.6, 0.5)	0.924
**Role emotional**						
Multivariate adjusted model (1)	0 (ref.)	1.8 (-0.2, 3.8)	2.3 (0.2, 4.5)	3.0 (0.7, 5.2)	2.0 (-0.7, 4.7)	0.099
Additionally adjusted for MD (2)	0 (ref.)	1.9 (-0.2, 3.8)	2.3 (0.1, 4.4)	3.0 (0.7, 5.3)	2.1 (-0.6, 4.9)	0.099
**Mental health**						
Multivariate adjusted model (1)	0 (ref.)	0.3 (-0.7,1.2)	0.7 (-0.3, 1.6)	0.5 (-0.4, 1.5)	-0.7 (-1.9, 0.6)	0.520
Additionally adjusted for MD (2)	0 (ref.)	0.3 (-0.7, 1.2)	0.6 (-0.4, 1.6)	0.5 (-0.5, 1.6)	-0.7 (-1.9, 0.7)	0.530

	**Baseline trans unsaturated fatty acid intake**					

Energy-adjusted TFA intake (g/day) (median)	0.4	0.8	1.0	1.2	1.7	
**Vitality**						
Multivariate adjusted model (1)	0 (ref.)	-1.3 (-2.4, -0.2)	-0.6 (-1.8, 0.5)	-0.2 (-1.8, 0.6)	-2.0 (-3.4, -0.6)	0.056
Additionally adjusted for MD (2)	0 (ref.)	-1.2 (-2.3, -0.1)	-0.4 (-1.6, 0.7)	0.1 (-1.1, 1.3)	-1.6 (-3.0, -0.2)	0.162
**Social functioning**						
Multivariate adjusted model (1)	0 (ref.)	-0.4 (-1.4, 0.6)	-0.2 (-1.3, 0.8)	-0.2 (-1.3, 0.8)	-1.7 (-2.9, -0.5)	0.013
Additionally adjusted for MD (2)	0 (ref.)	-0.5 (-1.5, 0.6)	-0.2 (-1.3, 0.8)	-0.2 (-1.3, 0.8)	-1.7 (-2.9, -0.4)	0.017
**Role emotional**						
Multivariate adjusted model (1)	0 (ref.)	0.2 (-1.8, 2.2)	0.3 (-1.8, 2.4)	0.4 (-1.8, 2.4)	-3.5 (-6.0, -1.0)	0.006
Additionally adjusted for MD (2)	0 (ref.)	0.1 (-1.9, 2.1)	0.2 (-1.9, 2.3)	0.4 (-1.8, 2.6)	-3.5 (-6.0, -1.0)	0.007
**Mental health**						
Multivariate adjusted model (1)	0 (ref.)	-0.3 (-1.3, 0.6)	-0.1 (-1.1, 1.0)	1.0 (-0.1, 2.1)	-0.2 (-1.4, 1.1)	0.646
Additionally adjusted for MD (2)	0 (ref.)	-0.3 (-1.3, 0.7)	-0.1 (-1.2, 0.9)	0.9 (-0.2, 2.0)	-0.2 (-1.4, 0.7)	0.634

The SUN cohort 1999-2010.

**Vitality: **vitality and energy feelings against tiredness or exhaustion

**Social Functioning**: grade in which mental health problems can interfere with habitual social life

**Role Emotional**: grade in which mental health problems can interfere with work activity or with other daily activities

**Mental Health**: general mental health including depression, anxiety, emotional and behaviour control and general positive effect

(1): Model adjusted for age, sex, smoking (non smoker, current, ex-smoker and missing value), leisure time physical activity (in quintiles of METs-h/wk score), total energy intake (Kcal/day), and baseline BMI (Kg/m^2^), MUFA, PUFA and, additionally, SFA (TFA intake) or TFA (SFA model).

(2): Model 1 additionally adjusted for adherence to the Mediterranean Diet score (0-8) (excluding SFA/MUFA ratio).

Multivariate regression coefficients and their 95% CI for the physical domains of the SF-36 according to baseline SFA and TFA intake (in quintiles) are shown in Table 3.

**Table 3:** Regression coefficients (b) and 95% Confidence Intervals (CI) for the association between baseline saturated (SFA) and trans unsaturated fatty (TFA) acid intake and the SF-36 physical domains after 4-y follow-up.

	Baseline saturated fatty acid intake					
SF-36 physical scores after 4 years of follow-up	Q1	Q2	Q3	Q4	Q5	p linear trend
Energy-adjusted SFA intake (g/day) (median)	22.8	29.7	33.5	37.3	45.5	
**Physical functioning**						
Multivariate adjusted model (1)	0 (ref.)	0.2 (-0.5, 0.8)	-0.04(-0.7, 0.6)	-0.6 (-1.3, 0.1)	-1.0 (-1.9, -0.2)	0.520
Additionally adjusted for MD (2)	0 (ref.)	0.3 (-0.4, 0.9)	0.1 (-0.5, 0.8)	-0.3 (-1.0, 0.4)	-0.7 (-1.5, 0.2)	0.068
**Role physical**						
Multivariate adjusted model (1)	0 (ref.)	0.1 (-1.8, 2.0)	0.8 (-1.2, 2.8)	1.1 (-1.0, 3.2)	-1.7 (-4.2, 0.9)	0.384
Additionally adjusted for MD (2)	0 (ref.)	0.3 (-1.5, 2.2)	1.2 (-0.8, 3.2)	1.6 (-0.6, 3.7)	-1.1 (-3.7, 1.5)	0.676
**Bodily pain**						
Multivariate adjusted model (1)	0 (ref.)	0.1 (-1.3, 1.6)	0.4 (-1.1, 2.1)	0.4 (-1.2, 2.0)	-0.6 (-2.5, 1.3)	0.664
Additionally adjusted for MD (2)	0 (ref.)	0.3 (-1.2, 1.7)	0.7 (-0.9, 2.3)	0.9 (-0.8, 2.5)	0.03 (-2.1, 2.0)	0.831
**General health**						
Multivariate adjusted model (1)	0 (ref.)	0.2 (-0.9, 1.3)	-0.1 (-1.2, 1.1)	-0.5 (-1.8, 0.8)	-1.6 (-3.1, -0.1)	0.027
Additionally adjusted for MD (2)	0 (ref.)	0.3 (-0.8, 1.5)	0.3 (-1.0, 1.5)	-0.02 (-1.3, 1.3)	-1.0 (-2.5, 0.6)	0.206

	**Baseline transunsaturated fatty acid intake**					

Energy-adjusted TFA intake (g/day) (median)	0.4	0.8	1.0	1.2	1.7	
**Physical functioning**						
Multivariate adjusted model (1)	0 (ref.)	-0.4 (-1.0, 0.2)	0.2 (-0.5, 0.8)	-0.1 (-0.8, 0.6)	-0.8 (-1.5, 0.02)	0.111
Additionally adjusted for MD (2)	0 (ref.)	-0.4 (-1.0, 0.2)	0.2 (-0.4, 0.9)	-0.02 (-0.7, 0.7)	-0.6 (-1.4, 0.2)	0.289
**Role physical**						
Multivariate adjusted model (1)	0 (ref.)	-0.8 (-2.6, 1.1)	0.09 (-1.9, 2.0)	-1.0 (-3.0, 1.1)	-2.3 (-4.6, 0.04)	0.050
Additionally adjusted for MD (2)	0 (ref.)	-0.7,(-2.5, 1.2)	0.3 (-1.7, 2.2)	-0.8 (-2.8, 1.3)	-2.0 (-4.4, 0.3)	0.085
**Bodily pain**						
Multivariate adjusted model (1)	0 (ref.)	-1.9 (-3.4, -0.5)	-1.2 (-2.7, 0.3)	-1.8 (-3.4, -0.2)	-2.6 (-4.4, -0.8)	0.017
Additionally adjusted for MD (2)	0 (ref.)	-1.9 (-3.4, -0.5)	-1.1 (-2.6, 0.4)	-1.6 (-3.2, -0.1)	-2.3 (-4.1, -0.5)	0.042
**General health**						
Multivariate adjusted model (1)	0 (ref.)	-0.03 (-1.2, 1.1)	1.3 (0.1, 2.5)	0.6 (-0.6, 1.9)	-1.1 (-2.5, 0.3)	0.161
Additionally adjusted for MD (2)	0 (ref.)	0.02 (-1.1, 1.1)	1.5 (0.3, 2.7)	0.9 (-0.4, 2.1)	-0.8 (-2.2, 0.6)	0.367

The SUN cohort 1999-2010.

**Physical Functioning**: grade in which health problems can interfere with physical activities such as walking, self-care or weight lifting

**Role Physical**: grade in which physical health problems can interfere with work activity or with other daily activities

**Bodily Pain**: pain intensity and its effect on work inside and outside home

**General Health**: personal belief regarding current health and future perspectives regarding health

(1): Model adjusted for age, sex, smoking (non smoker, current, ex-smoker and missing value), leisure time physical activity (in quintiles of METs-h/wk score), total energy intake (Kcal/day), and baseline BMI (Kg/m^2^), MUFA, PUFA and, additionally, SFA (TFA intake) or TFA (SFA model).

(2): Model 1 additionally adjusted for adherence to the Mediterranean Diet score (0-8) (excluding SFA/MUFA ratio).

For SFA intake, a significant inverse association was found for physical functioning, and general health domains. For the general health domain: (Q5 vs. Q1), b = -1.6; 95% CI = -3.1 to -0.1. General health also showed a statistically significant dose -response relationship (p for trend < 0.05). For TFA, an inverse association was found for bodily pain: (Q5 vs. Q1), b = -2.6; 95% CI = -4.4 to -0.8 with a dose-response relationship (p for trend < 0.05).

When we repeated the analysis additionally adjusting for the adherence to a Mediterranean diet score, the association remained significant for TFA intake and the mental domains and bodily pain.

No association was observed between n-3 PUFAs, n-6 PUFAs, MUFA or the ratio n-3 *vs*. n-6 intake and the SF-36 domains. Neither was found for seed oils, butter, margarine or olive oil (data not shown).

## Discussion

The present study showed a harmful association between the highest intake of TFA and several SF-36 domains. The association remained significant for the mental domains (except for mental health), and bodily pain after controlling for potential cofounders including the adherence to the Mediterranean diet. An inverse association for SFA intake and some of the physical domains was also found, although after adjusting for the adherence to the Mediterranean diet score this relationship was not statistically significant.

These associations could mean that the physiological changes that occur when this kind of fatty acid is consumed could influence mostly the mental quality of life and therefore the self perception of "well being". So, the participants with the highest intake would perceive themselves more tire and worn out, with social and role disability due to emotional problems, and with severe limiting pain than the participants with the lowest TFA intake. Although these are perceived health measures rather than biological measures, self-related heath status has been shown to be a powerful predictor of mortality at long term [2].

On the other hand, for the mental quality of life domains, the magnitude of the differences between the lowest and highest quintile of TFA intake were about 1.5-3 points. There is a debate on how to define meaningful differences on the SF-36 scores in a clinical setting. Changes in 3-, 5-, and 10- points have been suggested as being clinically significant for clinical populations [37]. Given the characteristics of our cohort that did not include patients, but healthy and relatively young adults, the practical significance of these differences could be even higher. Although few studies have examined this issue directly, several investigators have raised the question of whether individuals with more severe impairments in HRQOL require a greater change to be considered meaningful than those with less severe impairments [38].

Fatty acids of trans configuration come from two main different sources: the major source is derived from industrially produced partially hydrogenated fat used in margarines, commercial cooking, and manufacturing processes (60% of the fats), and smaller amounts are naturally present in dairy and meat products from ruminants (6% of the fats) [39]. Based on the evidence to date, TFA intake, particularly the industrial trans-18:2 isomer, is associated with substantial risk of coronary heart disease (CHD) [40-43]. The adverse effects of TFA on CVD are thought to be mediated by increases in plasma concentrations of LDL-cholesterol, reductions in HDL-cholesterol, pro-inflammatory changes, endothelial dysfunction, and possibly by insulin resistance and displacement of essential fatty acids from membranes [44,45].

The relation of TFA intake to other disease outcomes has been examined less extensively than for CHD. However, emerging evidence suggests that TFA acid intake may influence additional non -lipid related pathways and outcomes. These include effects on systemic inflammation, endothelial dysfunction, visceral adiposity, insulin resistance, and arrhythmic risk [46]. Moreover, TFA intake has been linked to accelerated cognitive decline in older adults [47], and higher risk of Alzheimer [48], and depression [49]. The harmful effect of TFA intake in these neuropsychological disorders supports our results which suggest that TFA intake specially affects mental quality of life. A possible explanation for our finding is that TFA promote endothelial dysfunction and increase the production of pro-inflammatory cytokines that may interfere with neurotransmitter metabolism and inhibit Brain-derived neurotrophic factor (BDNF) expression among other physiological effects [50,51]. BDNF is a peptide critical for axonal growth, neuronal survival and synaptic plasticity and function. Therefore, it is likely that the consumption of foods containing TFA in their composition could increase the vulnerability to some mental or neurological disorders or act negatively on mental quality of life.

Although our results suggest a detrimental role of TFA on mental quality of life, our findings are modest. The present study was carried out among a sample in which TFA intake was very low (the median of intake was 1 gram per day). This intake was lower than the median intake for Spanish population which is 2.1 grams per day, [52] and far away from the higher consumption corresponded to the United States and Canada with values of 3-4 grams per day [53]. Therefore, the repercussion of these findings might be really important in these populations where the consumption is very high comparing to our cohort and where the main sources of TFA are artificial foods [54].

Several limitations in our study have to be addressed. Diet was ascertained at baseline and quality of life after 4-years of follow-up, therefore we acknowledge that baseline scores of quality of life were unknown. Consequently, in spite of the fact that the follow-up of participants allows a sufficient long induction period, it could still be possible to speculate that a poor-quality diet may be a result of mental health symptoms, rather than a causal factor.

We acknowledge that although the food frequency questionnaire has been validated using dietary records as gold standard, this is not the best method to validate some dietary fatty acids intake such n-3 PUFAs. The use of a validation method with biomarkers as gold standard is recommended. This could lead to certain, probably non-differential, misclassification bias in the dietary n-3 PUFAs assessment.

Another fact to take into account is that quality of life is a complex concept with various dimensions. Nevertheless, the use of the SF-36 questionnaire for evaluating the physical and mental dimensions of quality of life is generally accepted, and its validity and reliability have been demonstrated in many population-based studies [55].

Some strengths of our study also deserve to be mentioned. They include its large sample size, its long-term follow-up, the multiple adjustments of our estimates for a variety of major potential confounders, the existence of published validation studies of our assessments, and the restriction to highly educated participants, which provides a better validity to the self-reported data.

## Conclusion

In summary, our findings suggest that TFA is the fat subtype that adversely affects quality of life. This association was stronger for the mental domains than for the physical domains of the SF-36. However, replication of these findings in prospective studies, including also a baseline ascertainment on quality of life scores is required in order to confirm the reported associations and their direction.

## List of abbreviations

SUN: Seguimiento Universidad de Navarra; SFA: Saturated fatty acids; PUFA: Polyunsaturated fatty acids; n-3 PUFAs: omega 3 polyunsaturated fatty acids; n-6 PUFAs: omega 6 polyunsaturated fatty acids; TFA: Trans unsaturated fatty acids; MUFA: Monounsaturated fatty acids; CI: Confidence intervals; Q5: 5^th ^quintile; Q1: 1^st ^quintile; OO: Olive oil; CVD: Cardiovascular diseases; BMI: Body mass index; MET: Metabolic Equivalent Time; HRQOL: Health related quality of life; CHD: coronary heart disease; BDNF: Brain derived neurotrophic factor.

## Competing interests

The authors declare that they have no competing interests.

## Authors' contributions

CR and AS-V participated in the planning and conception of the research questions and the study design, contributed on the process of hypothesis generation, data collection, statistical analyses and manuscript preparation. AS-V was the principal investigator of the study and primarily conceptualized the research. CR and PH carried out the data retrieval, statistical analyses, and manuscript drafting. CR drafted the article, and all authors participated in interpreting the data and critically revising the manuscript for important intellectual content. All authors read and approved the final manuscript.

## Authors' information

CR is a PhD student at the Department of Clinical Science, University of Las Palmas de Gran Canaria. She is a member of the Nutritional Research group of this university and works as a researcher in the project: "The role of diet and physical activity on quality of life and mental illness in the SUN project". Supported by the Spanish Government (Instituto de Salud Carlos III, Fondo de Investigaciones Sanitarias; grant PI080819).

PH is an Associate Professor of Preventive Medicine and Public Health at the Department of Clinical Science, University of Las Palmas de Gran Canaria, Spain. She belongs to the Nutritional Research group of this University.

MB-R is an Associate Professor at the Department of Preventive Medicine and Public Health, University of Navarra, Spain. She is a member of the Scientific Committee of the SUN project.

MR-C is an Associate Professor at the Department of Preventive Medicine and Public Health, University of Navarra, Spain, and also a researcher in the SUN project.

CL is an Assistant Professor at the Department of Preventive Medicine and Public Health, University of Navarra, Spain, and also a researcher in the SUN project.

AS-V is an Associate Professor of Preventive Medicine and Public Health at the Department of Clinical Science, University of Las Palmas de Gran Canaria, Spain. She belongs to the Nutritional Research group of this University and she is the principal researcher of the project: "The role of diet and physical activity on quality of life and mental illness in the SUN project". Supported by the Spanish Government (Instituto de Salud Carlos III, Fondo de Investigaciones Sanitarias; grant PI080819).
